# Natural Products as a Source of Anti-Inflammatory Agents Associated with Inflammatory Bowel Disease

**DOI:** 10.3390/molecules18067253

**Published:** 2013-06-19

**Authors:** Trishna Debnath, Da Hye Kim, Beong Ou Lim

**Affiliations:** Department of Life Science, College of Biomedical & Health Science, Research Institute of Inflammatory Disease, Konkuk University, Chungju 380-701, Korea

**Keywords:** natural products, inflammatory bowel disease, inflammation, cytokines, reactive oxygen species

## Abstract

Accumulating epidemiological and clinical study indicates that inflammation is a significant risk factor to develop various human diseases such as inflammatory bowel disease (IBD), chronic asthma, rheumatoid arthritis, multiple sclerosis, and psoriasis. Suppressing inflammation is therefore important to control or prevent various diseases. Among them, IBD is one of the major problems affecting people worldwide. IBD affects at least one in a thousand persons in many Western countries. Various natural products have been shown to safely suppress pro-inflammatory pathway and control IBD. *In vivo* and/or *in vitro* studies indicate that anti-IBD effects of natural products occur by inhibition of the expression of pro-inflammatory cytokines (for example, tumor necrosis factor-α (TNF-α), intercellular adhesion molecule expression and pro-inflammatory mediators (such as inducible nitric oxide synthase (iNOS) and cyclooxygenase 2 (COX2), master transcription factors (such as nuclear factor-κB (NF-κB)), reactive oxygen species (ROS) and by improving the antioxidant activity. In this review, we summarize recent research focused on IBD and the effects that natural products have on IBD factors.

## 1. Introduction

Inflammation is the first biological response of the immune system to infection or irritation. The word ‘inflammation’ comes from the Latin “inflammo,” meaning “I set alight, I ignite.” A verity of stimuli such as physical damage, ultra violet irradiation, microbial invasion, and immune reactions are responsible for inflammation. Inflammation is characterized by redness, heat, swelling, and pain. Based on timing and pathological features, there are two major categories available for inflammation: acute and chronic. Chronic inflammatory disease is characterized by persistent inflammation. On the other hand, acute inflammation occurs over seconds, minutes, hours, and days. 

IBD is inflammation within the gastrointestinal (GI) tract characterized by chronic or relapsing immune system activation. There are two types of IBD: ulcerative colitis and Crohn’s disease [1]. Ulcerative colitis occurs in the inner lining of the colon (large intestine) or rectum and the common symptoms are diarrhea, abdominal cramps and rectal bleeding while those with Crohn’s disease, experience pain in the abdomen, especially in the lower right side with symptoms—diarrhea, fatigue, weight loss and occasionally bleeding - and occurs in the deep layers of the intestinal wall. 

Generally, anti-inflammatory drugs or immunosuppressive drugs such as 5-aminosalicylic acid (5-ASA) and 6-mercaptopurine are used to treat IBD; steroids and non-steroidal anti-inflammatory drugs are effective for temporary relief of symptoms. However, drug-induced severe side effects occur, and most of these treatments are inadequate. [2,3,4]. Therefore, it is crucial to identify a new and safe drug for preventing or treating IBD [5]. Consequently, many people with IBD turn to alternative medicine including traditional plant based remedies [2].

A number of studies reported that plant-derived extracts or plant derivatives such as phenolic compounds and flavonoids show anti-inflammatory activity by controlling the levels of various inflammatory cytokines or inflammatory mediators including IL-1, IL-6, IL-10, TNF-α, NF-κB, NO, iNOS and COX-2. Moreover, many crude extracts and chemical constituents of plants have pharmacologic effects and clinical benefits. However, the claims of benefits of many plants or plant based medicines marketed to the general population are only supported by empirical or preliminary scientific data [6]. 

Therefore, the aim of this review is to provide an overview of the effects of more than 50 natural products used worldwide for the treatment of IBD. For this purpose, electronic databases including Pubmed, Scopus, Embase, and Google Scholar were searched for each of the natural products, and all retrieved plants sited here were examined by *in vitro*, *in vivo*, or clinical trials to determine the their efficacy on IBD or related factors.

## 2. The Factors of IBD

The most agreed upon hypothesis currently implicates a combination of one or more factors such as: immune deregulation which is caused by genetic or environmental factors, abnormal gastrointestinal (GI) tract luminal factors (e.g., microorganisms constituting the GI tract flora), oxidative stress, and defects in the GI mucosal barrier that allow luminal factors to penetrate the mucosa [7,8]. However, the specific etiology of IBD is unclear [9].

### 2.1. Abnormal Immune Response

Defective responses in both the innate and the adaptive immune systems in IBD are reported [10]. The character of the cells mediating innate immunity such as neutrophils, macrophages, dendritic cells, and natural killer cells are changed and abnormal mucosal T helper (Th) cell response and over expression of cytokines such as TNF-α, interferon-γ (IFN-γ), interleukin-1β (IL-1β), IL-12 as well as IL-6 were found in patients with IBD [11,12]. These changes cause destructive affections of the mucous membrane of the large intestine, e.g., impairment of mucous barrier, swelling, ulcers, erosions, and hemorrhages [13,14,15]. Cytokines are key signaling molecules of the intestinal immune system, which play an important role in IBD. TNF-α is one of the most important pro-inflammatory cytokines that directly influence intestinal epithelial tissue and Interleukin-1ß and IL-6 are also key mediators of IBD progression [2]. Excessive TNF-α expression results in damaging the epithelial barrier, initiation of apoptosis in epithelial cells, and initiation of chemokine secretion by colonic epithelial cells [2]. In addition, Liu and Wang reported that TNF-α that is released from macrophages during the first stage of inflammation plays a vital role in 2,4,6-trinitrobenzenesulfonic acid (TNBS)—induced colitis and is identically the key controller of the inflammatory flow in this IBD model [16]. An IL-1 receptor antagonist was found to decrease the permeation of inflammatory cells into the large intestine, the MPO activity of cells in areas of edema, and colon necrosis in acute colitis model [17]. Interleukin-6 is related to necrosis in the colon that leads to tissue destruction. Several investigators have reported anti-inflammatory properties of different types of plants or natural compounds by reducing the cytokine expression on *in vitro* and *in vivo* model. For example, *Inonotus obliquus*, grown on germinated brown rice, showed anti-IBD effect and reduced the pro-inflammatory cytokine expression in mice [1]. *Inonotus obliquu*, *Coriolus versicolo* and *Prunus mume* extracts have been found to have anti-IBD effect which likely occurred by the attenuating proinflammatory gene expression, particularly that of TNF-α, IL-4, and IL-1ß [18,19,20].

### 2.2. Reactive Oxygen Species

Oxidative stress is one of most important etiological and/or triggering factor for IBD. The damaging effects of ROS have been well known in the inflammatory process [21,22,23]. ROS and Reactive nitrogen species (RNS) metabolites are associated with the initiation and progression of IBD [24]. ROS are very unstable because of their high reactivity that occur peroxidation and the oxidative damage to DNA and proteins [25]. Recent studies have found decreased total antioxidant activity and increased ROS in patients with IBD [22,23,26,27]. It was demonstrated that the antioxidant activity of some herbs can improve IBD. 

### 2.3. Microbial Effect

The roles of microbial content of the GI tract in IBD have already been reported [28]. The interactions between the host susceptibility, mucosal immunity and intestinal microflora are responsible for the pathogenesis of IBD. The composition of the flora in an individual is stable in the human body, but differs between the stomach and upper bowel, lower small bowel, right colon and rectum. Moreover, the flora recovered from feces is also different from mucosa-associated or intraepithelial flora [28]. The resident microbiota plays a critical role in modulating the immune response of the gut as well as in the initiation and perpetuation of IBD. Compared to normal, the higher concentration of intestinal bacteria was shown in IBD that increases progressively with disease severity [29]. Therefore, adjusting the gut bacterial flora by antibacterial agents may be beneficial to treat UC [30,31]. Currently, the prebiotics used in the functional food industry are oligosaccharides including lactulose, fructo-oligosaccharides, inulin, galacto-oligosaccharides. The consumption of prebiotics may influence the activity and composition of colonic microbiota. Therefore, a great interest of finding the various prebiotics or mixtures of prebiotics that could play a biological activity throughout the whole colon and reduce the rate of chronic colonic diseases such as Crohn’s disease and colorectal cancer. In this regard, specific studies have been carried out to identify potential key ingredients in various medicinal plants known for their ancient uses as healing remedies [32]. The recent study showed that two commercially-available natural polysaccharide supplements modulated microbial community in the different areas of the colon [33].

### 2.4. Nuclear Factor-Kappa B

Nuclear factor-kappa B (NF-kß) protein promotes the expression of more than 150 genes, many of them act as important roles in the regulation of inflammation and programmed cell death (apoptosis) [34,35]. Over expression or inappropriate activation of NF-kB has been shown in human IBD [36,37]. However, the activation of NF-kB is not causative for IBD, but NF-KB seems to play an important role in these diseases [34]. Thus, the inhibitors of NF-kB or IKK can be used as a treatment strategy for managing IBD.

### 2.5. iNOS and COX-2

Under a physiological state, nitric oxide is synthesized from l-arginine with the involvement of constitutive NO synthases—eNOS and nNOS. Large amount of NO-synthases, especially iNOS is indicative of ulcerative colitis [38]. Prostaglandins produced by COX-1 and COX-2, that is another important system involved in the development of ulcerative colitis. Under physiological conditions, COX-1 of mRNA is expressed in the large intestine. At the same time, COX-2 also appears in the interstitial tissue, epithelial cells, and neurons of sub-mucous and inter-muscular plexuses. Increasing amounts of prostaglandins synthesized by COX-2 are involved in the inflammatory process [39].

## 3. Modern Evidence for the Efficacy of Plants on IBD

### 3.1. Coriolus versicolor

*Coriolus versicolor* is a mushroom belonging to the class Homobasidiomycetes. The ethanol extracts from *Coriolus versicolor* showed anti-IBD effects. Colitis was induced in male BALb/c mice by giving them drinking water containing dextran-sulfate sodium (DSS). *Coriolus versicolor* treatment reduced the expression of pro-inflammatory cytokine profile such as TNF-α, IL-1β and IL-6. It showed a significant reduction in the expression of signal transducers and activators of transcription (STAT)1 and STAT6 molecules and lower IFN-γ and IL-4 expression. The higher immunoglobulin (Ig)A and lower IgE levels were found in serum of the DSS + *Coriolus versicolor* treated group mice compared to DSS treated group [19].

### 3.2. Brahmi

Yamada *et al.* conducted a comparative study of the immunostimulatory effects of the medicinal herbs Echinacea, Ashwagandha and Brahmi [40]. Among them, Ashwagandha (*Withania somnifera*) and Brahmi (*Bacopa monnieri*) are commonly found in India. They compared the immunostimulatory effects of Ashwagandha and Brahmi with the effect of Echinacea and observed the Brahmi diet enhance immune function by increasing the levels of IgA and IgG in the serum of male Sprague Dawley rats.

### 3.3. Inonotus obliquus

*Inonotus obliquus* (IO) is a mushroom belonging to the inonotus genus and Hymenochaetaceae family of Basidiomycetes living as a parasite on birches in Europe and Asia. In Russia, the black, shapeless overgrowth of the birch is usually called chaga [41]. The water extracts of IO suppressed the ulcerative colitis in DSS induced male BALB/c mice [18]. The water extracts decreased the expression of TNF-𝛼, IL-4 and STAT1, STAT6 compared to those of the disease group. It also showed the inhibitory activity on LPS induced TNF-𝛼, STAT1, pSTAT1, STAT6, and pSTAT6 production in RAW264.7 cells. IO grown on germinated brown rice (IOGBR) ethanol extracts showed potent activity on ulcerative colitis in mice [1]. IOGBR reduced proinflammatory mediators such as tumor necrosis factor (TNF)-*α*, cyclooxygenase (Cox)-2, interleukin (IL)-4, interferon (IFN)-γ and decreased STAT1 and STAT6 expression. Immunoglobulins (Ig) act as an important role in inflammation. Therefore, IOGBR extracts suppressed the expression of IgE and IgA in the spleen and mesenteric lymph node (MLN) compared to those of the DSS-induced colitis group. The extracts suppressed the pathogenic shortening of colon length and reduced DSS-induced colonic tissue destruction. 

### 3.4. Cordyceps sinensis

*Cordyceps sinensis* is a fungus belonging to the family Clavicipitaceae. The water extracts of *Cordyceps sinensis* was applied to the C57Bl/6N mice to evaluate the effects of immune function of mesenteric lymph node (MLN) lymphocytes. The results indicated that the IgE concentrations in serum and MLN lymphocytes were significantly lower in *C. sinensis*-treated mice than in the control mice. The extracts increased the proportion of CD4(^+^) and CD8(^+^) T cells in MLN lymphocytes [42]. Different research found that *Cordyceps militaris* extract suppresses dextran sodium sulfate-induced acute colitis in BALB/c mice by suppressing disease symptoms such as body weight loss, diarrhea and gross bleeding. The extracts prevented shortening of the colon and crypt length and the epithelial damage [43].

### 3.5. Prunus mume

*Prunus mume* Sieb. et Zuce belongs to the genus Prunus and family Rosaceae. This fruit known as Ou-mae and has been used in Korea as a folk medicine to treat fever, cough and intestinal disorders [44]. In addition, the unripe *P. mume* showed inhibitory activity against *Helicobacter pylori* motility [45]. Yingsakmongkon *et al.* studied concentrated fruit juice to prevent or reduce the virus infection in human influenza [46]. Prunate isolated from *P. mume* has inhibitory activity on the proliferation of cancer cells [46,47]. Antioxidant activities of flowers and fresh fruits of *P. mume* have been examined [48,49]. *Prunus mume* mixture treatment decreased the expression of TNF-α, COX-2, IL-4, STAT6, INF-γ, STAT1 in mice with DSS-induced colitis [20]. In addition, Mume Fructus is the fruit of *Prunus mume.* The mature green fruit is heated at a low temperature until the yellowish colored pulp changes to brown. Then, the peel wrinkles and is braised until it turns black. The carbonized dried form is preserved and used. The water extracts of Mume Fructus showed 1,1-diphenyl-2-picrylhydrazyl (DPPH), 2,2'-azino-bis(3-ethylbenzthiazoline-6-sulfonic acid) (ABTS), hydroxyl (OH^•^), superoxide (O_2_^−•^) radicals and nitrite scavenging activities, inhibition of linolic acid oxidation and reducing power activity [50]. Mume Fructus pill (FMP) has been used as a folk medicine in China. For the treatment of gastrointestinal disorders, Mume Fructus pill was approved by the State Food and Drug Administration (SFDA) of China in 2001 (Approval No. Z11021100). From the study of Liu *et al.* FMP prevented diarrhea, colon weight increase, colonic accretion, ulceration and myeloperoxidase (MPO) activity elevation [51]. The FMP recovered colonic damage and promoted abnormal cytokine secretion in rats with colitis. 

### 3.6. Gardenia jasminoides

*Gardenia jasminoides* Ellis (GJE) is a flowering plant which belongs to the genus Gardenia and family Rubiaceae. It has traditionally been used as folk medicine in many Asian countries. The ethanol and water extracts from *Gardenia jasminoides* Ellis have been researched to evaluate their antioxidant activity. Both extracts showed high antioxidant activity by scavenging various radicals. The extracts showed strong reducing power, nitrite scavenging activity, linoleic acid oxidation inhibition, superoxide dismutase-like (SOD-like) activity, and catalase activity *in vitro* [52]. Glycoprotein isolated from *Gardenia jasminoides* Ellis (GJE) fruits suppressed MPO activity, TBARS level, and NO production and inhibited the over production of iNOS, COX-2, and NF-kappa-β (p50) in DSS-induced mice [53]. 

### 3.7. Chrysanthemum indicum

*Chrysanthemum indicum* Linné is an herb that belongs to the genus of chrysanthemum and family of Asteraceae. It has traditionally been used as folk medicine in China and Korea and treats various immune-related disorders, hypertension and various infectious diseases including pneumonia, colitis, stomatitis, carbuncle and fever [54,55]. Butanol-soluble fraction of *Chrysanthemum indicum* inhibited on the auricle edema in mice [54]. 

### 3.8. Benincasa hispida Cogn.

Waxgourd (Benincasa hispida Cogn.) belongs to a family of Cucurbitaceae and has been used in traditional Chinese medicine to treat inflammation and high blood pressure. It is good for mineral detoxification, lowering fever and strengthening the function of the bladder and small and large intestines [56]. The seed extracts of *Benincasa hispida* inhibits the histamine secretion and show antitumor effects by enhancing immunoreactions [57]. Different parts of the wax gourd such as the peel, core and pulp as well as fresh seeds have antioxidant capacity [58]. Water extract from dry seeds of *Benincasa hispida* showed strong antioxidant activity by scavenged DPPH, ABTS and hydroxyl radical in a dose-dependent manner [59]. The extracts also showed inhibitory activity on linoleic acid oxidation and nitrite radical [59]. The dried seed extracts produced significant reduction in ulcer index in Wistar albino rats. Further, the extracts reduced MDA content along with increasing CAT levels when compared to the control group [60].

### 3.9. Avicennia marina

*Avicennia marina* (*A. marina*) is a plant of the Acanthaceae, commonly known as grey mangrove or white mangrove. *A. marina* decreased the colonic lipid peroxides, glutathione peroxidase, and serum nitric oxide, lesion score and wet colon weight, and increased the colonic and erythrocyte superoxide dismutase and glutathione levels compared with colitis control [61].

### 3.10. *Patrinia scabiosaefolia*

*Patrinia scabiosaefolia* Fisch belongs to the family Valerianaceae. In Asia, the plants are usually used to treat anti-inflammatory diseases, especially for colonic inflammations, virus infections, hepatitis, and uteritis [62]. The root extracts of *Patrinia scabiosaefolia* Fisch suppressed weight loss, diarrhea, gross bleeding, infiltrations of immune cells, prevented shortening of colon length and enlargement of spleen size, down regulated the abnormal secretions and mRNA expressions in mice with DSS induced colitis [2]. Histological study indicated that the extracts reduced edema, mucosal damage, the loss of crypts [2].

### 3.11. *Ficus bengalensis* Linn.

*Ficus bengalensis* Linn. from the family Moraceae is a reputed plant in Ayurvedic medicine. In Ayurvedic literature, it is known as “banayan tree.” The milky juice from the stem, seeds, or fruit of this plant is used externally for rheumatism and on the soles of feet when inflamed. It is also used for the treatment of dysentery and diarrhea. The ethanol extracts from the bark of this tree declined colon mucosal damage index and disease activity index and decreased the MPO, MDA, NO, and increased the SOD activity in the colons of rats with IBD [63]. 

### 3.12. Ginger *(Zingiber officinale)*

Ginger, belongs to the family Zingiberaceae, and its component zingerone were investigated to determine its anti-inflammatory activity in mice colitis induced by TNBS. They ameliorated TNBS-induced colonic injury in a dose-dependent manner. Their pathway investigation on gene expression profiles has been found to control cytokine-related pathways significantly. They suppressed TNBS-induced NF-κB activation and IL-1β protein level in the colon [64]. 

### 3.13. *Withania somnifera*

*Withania somnifera* (Dunal), belongs to the family Solanaceae, is used as a medicine since 2500 years in Indian medicinal classic “Ayurveda”. Aqueous extract of its the root showed anti-oxidant activity by reducing H_2_O_2_ and NO. It has lipid peroxidation inhibition activity. The extracts scored positively on histopathological parameters like necrosis, edema, neutrophil infiltration in TNBS-induced IBD rat model [65].

### 3.14. *Garcinia cambogia*

*Garcinia cambogia*, known as Malabar tamarind, is native to Southeast Asia. Its fruit extracts have been suggested to have a variety of pharmacological properties including antiulcer activities. The anti-inflammatory activity of a garcinia extract was assessed in TNBS-induced colitis rats. The extracts treatment improved the macroscopic damage and reduced MPO activity, COX-2 and iNOS expression. It was also able to reduce PGE2 and IL-1β colonic levels. It did not show any mortality nor toxicity signals after oral administration [66]. 

## 4. *In vitro* Studies of Plant on IBD Related Factors

Scouring rush (*Equisetum hyemale L.*) is a perennial herb belong to the family Equisetaceae. [67]. Different extracts of this plant showed antioxidant and anti-inflammatory activities. The extracts have reducing power, metal-chelating activity, superoxide and nitrite scavenging abilities. The extracts showed inhibitory activity on NO, iNOS and COX-2 production in RAW 264.7 macrophages [67]. Schizonepeta tenuifolia is an herb belonging to the family of Lamiaceae. The methanol extracts from this plant showed inhibitory activity on iNOS in LPS-stimulated RAW 264.7 cells with highly antioxidant properties [68]. *Antrodia salmonea* T. T. Chang et W. N. Chou (Polyporaceae), a new species of the genus *Antrodia,* identified in 2004 [69]. Ethanol extracts from this plant showed nitric oxide (NO) production, expression of inducible nitric oxide synthase (iNOS) and COX-2 proteins inhibition and enzymatic antioxidant activity [70].

## 5. Conclusions

A great deal of evidence suggests that chronic inflammation promotes development of numerous human diseases, including IBD. Various herbal products have been used for the treatment of IBD. In this review we found that these natural products have shown their usefulness in IBD by different mechanisms of action such as inhibiting the production of NO, Cox-2, immunomodulatory properties, antimicrobial activities, antioxidant activities, and antiulcer properties which are summarized in detail in Table 1 and Table 2. As shown in Table 1, some of these plants showed only one or two mechanisms of action such as in *Bacopa monnieri,* *Vaccinium myrtillus,* *Coriolus versicolor,* *Inonotus obliquus.* However, in some of the plants, various mechanisms of action are shown. For example* Garcinia cambogia* and liquorice are effective in IBD for their pro-inflammatory cytokines and NO and Cox-2 inhibitory and antioxidant properties. Considering the devices of action of these plants, the mixture or combination of some of them may be beneficial due the numerous mechanisms involved in IBD. Based on the some *in vitro* studies, some plants are more effective to reduce IBD related factors. For example, *Taiwanofungus salmoneus* have antibacterial and NO, TNF-α scavenging activities, *Labisia pumila var. pumila, Labisia pumila var. alata, Labisia pumila var.* *lanceolata* have NO, antifungal, and anticancer activities, *Artemisia herba-alba, Ruta chalpensis L, Peganum harmala L* have anti-oxidant, anti-cancer, and anti-inflammatory activities. However, the exact mechanisms behind the anti-IBD effects of some of these natural products are still unclear. Therefore, additional *in vivo* research will be needed to determine their effects and to find which specific factors are involved in improving IBD in humans.

**Table 1:** *In vivo* studies on plants medicine for the treatment of inflammatory bowel disease.

Study	Plant	Part	Model	Species	Results
Debnath *et al.* [1]	*Inonotus obliquus* on germinated brown rice	Total mushroom	DSS	Mice	↓TNF-α, Cox-2, ↓STAT1, STAT6 ↓IFN-γ, IL-4 ↓IgE ↑IgA Prevented shortening of colon and crypt length and epithelial damage
Cho *et al.* [2]	*Patrinia scabiosaefolia*	Root	DSS	ICR mice	↓Weight loss, diarrhea, gross bleeding, infiltrations of immune cells. ↓TNF-α, IL-1β, and IL-6 mRNA level
Lim *et al.* [18]	*Coriolus versicolor*	Total mushroom	DSS	mice	↓TNF-α, IL-1β, IL-6 ↓STAT1, STAT6 ↓IFN-γ, IL-4 ↓IgE ↑IgA
Choi *et al.* [19]	*Inonotus obliquus*	Total mushroom	DSS	mice	↓TNF-α, ↓STAT1, STAT6
Jin *et al.* [20]	*Prunus mume*	Mixture	DSS	mice	↓TNF-α, Cox-2, ↓STAT1, STAT6 ↓IFN-γ, IL-4 ↓IgE ↑IgA Prevented shortening of colon and crypt length and epithelial damage
Yamada *et al.* [40]	*Bacopa monnieri* (Brahmi)	Herb		rat	↓IgA and IgG in the serum
Park *et al.* [42]	*Cordyceps sinensis*	Mushroom		C57Bl/6N mice	↓IgE in serum and MLN ↑CD4(^+^) and CD8(^+^) proportion in MLN.
Han *et al.* [43]	*Cordyceps militaris*	Mushroom	DSS	mice	Prevented shortening of colon and crypt length and epithelial damage.
Liu *et al.* [51]	Mume Fructus (*Prunus mume )*	Fruits	TNBS	Rat	↓diarrhea, colonic accretion, ulceration, ↑IFN-γ, IL-4
Rise *et al.* [61]	*Avicennia marina*	Plant	acetic acid	Mice	↓Colonic lipid peroxides, serum nitric oxide. ↑SOD and glutathione levels
Patel *et al.* [63]	*Ficus bengalensis*	Bark	TNBS	Wistar rats	↓MPO, MDA, NO ↓SOD
Hsiang *et al* [64]	*ginger (Zingiber officinale)*	Zingerone	TNBS	mice	↓NF-κB activity and IL-1β signalling pathway
Pawar *et al.* [65]	*Withania somnifera*	Root	TNBS	Rat	Positively scored on histopathological parameters, lipid peroxidation, H2O2 nad NO scavenging activities.
Rosillo *et al.* [71]	*Punica granatum*	Polyphenols	TNBS	Rat	↓iNOS, COX-2, p38, JNK, pERK1/2, IKBα and nuclear p65 NF-κB
Jagtap *et al*. [72]	*Bombax malabaricum*	Phytochemicals	indomethacin and iodoacetamide, acetic acid	Rat, mice	↓Ulcer score and MPO ↓TNF-α
Jung *et al.* [73]	Apples *(Malus spp)*	polyphenol			↓Proinflammatory gene expression
Dost *et al.* [74]	*Garcinia kola*	Herb	TNBS	Rat	↓Colonic damage ↑Antioxidant enzymes

TNBS: 2,4,6-Trinitrobenzene sulfonic acid; DSS: Dextran-sulfate sodium, IL: Interleukin; NF-κB: Nuclear factor κB; IFN: Interferon; NO: Nitric oxide; TNF-α: Tumor necrosis factor α; iNOS: Inducible nitric oxide synthase; Cox: Cyclooxygenase, MPO: myeloperoxide; Ig: Immunoglobulin; STAT: signal transducer and activator of transcription, ERK: Extracellular signalregulated kinase, JNK: c-Jun NH2-terminal kinase; PGE2: Prostaglandin E2; SOD: Superoxide dismutase; CAT: Catalase; MDA: Malondialdehyde.

**Table 2:** *In vitro* studies of plants medicine and their target on IBD-related factors.

Study	Plant	Part/extracts	Results
Debnath *et al.* [50]	*Mume Fructus (Prunus mume)*	Fruits	Antioxidant activity
Debnath *et al.* [52]	*Gardenia jasminoides Ellis*	Fruits	Antioxidant activity
Samad *et al.* [59]	*Benincasa hispida*	Seed	Antioxidant activity
Triebel *et al.* [75]	*Vaccinium myrtillus*	Plant	↓Pro-inflammatory mediators
Edmunds *et al.* [76]	*Actinidia chinensis* *Actinidia deliciosa*	Fruit	↓NO and cytokine secretion
Zia-Ul-Haq *et al.* [77]	*Black gram (Vigna mungo L.), Green gram (Vigna radiate) soybean (Glycine max.) lentil (Lens culinaris Medik)*	Seed	↓COX_2_, PGE_2_
Mueller *et al.* [78]	Allspice *(Pimenta officinalis)*	Fruit	↓COX2, TNF-alpha and IL-6 ↑IL-10
	Anise *(Pimpinella anisum)*	Fruit	
	Basil *(Ocimum basilicum)*	Leaves	
	Bay leaves *(Laurus nobilis)*	Leaves	
	Bilberry *(Vaccinium myrtillus)*	Phenols, (anthocyanins)	
	Black pepper *(Piper nigrum)*	Fruit	
	Cacao *(Theobroma cacao)*	Seed	
	*Caraway (Carum carvi)*	Seed	
	*Cardamom (Elettaria cardamomum)*	Seed	
	*Chili pepper (Capsicum annuum)*	Fruit	
	*Cinnamon (Cinnamomum cassia)*	Bark	
	*Clove (Syzygium aromaticum)*	Flower	
	*Coriandrum sativum*	Seed	
	*Zingiber officinale*	Rhizomes	
	*Lemon grass (Cymbopogon citratus)*	Leaves	
	*Licorice (Glycyrrhiza glabra)*	Root	
	*Marjoram (Origanum majorana)*	Leaves	
	*Nutmeg (Myristica fragrans)*	Fruits	
	*Oregano (Origanum onites)*	Leaves	
	*Paprika (Capsicum annuum)*	Fruits	
	*Rooibos tea (Aspalathus linearis)*	Leaves	
	*Rosemary (Rosmarinus officinalis)*	Plant	
	*Sage (Salvia officinalis)*	Leaves	
Jedinak *et al.* [79]	*Oyster mushroom (Pleurotus ostreatus)*	Mushroom concentrate	↓COX-2 and iNOS, ↓NF- κB, AP-1
Chiang *et al.* [80]	*Taiwanofungus salmoneus*	Mycelia	Antibacterial activity ↓NO, TNF-α
Ruangnoo *et al.* [81]	*Smilax corbularia*	Plant	↓NO, TNF-α and PGE2
Karimi * et al.* [82]	*Labisia pumila var. pumila Labisia pumila var. alata Labisia pumila var. lanceolata*	Leaves and roots	↓NO Antifungul, and anticancer activity
Khlifi *et al.* [83]	*Artemisia herba-alba*, *Ruta chalpensis L, Peganum harmala L.*	Leaves	Anticancer, Antioxidant (DPPH, ABTS radical scavenging activity) Anti-inflammatory activities (↓iNOS mRNA)
Jiménez-Estrada *et al.* [84]	*Krameria erecta*, *Struthanthus palmeri*, *Phoradendron californicum*, *Senna covesii Stegnosperma halimifolium*	Plant	Anti-oxidant and antiproliferative activities
Han *et al.* [85]	*Artemisia capillaris*	Capillarisin	↓TNF-α, IL-6, IL-1β, and PGE_2_ protein expression ↓COX-2 iNOS mRNA expression ↓ERK, JNK, and NF-κB
Choe *et al.* [86]	*Rhodiola sachalinensis*	Phenolic compounds from root	Anti-oxidant activity NO scavenging activity
Bang *et al.* [87]	*Achyranthes japonica*	Root	↓NO, iNOS, ERK, JNK, P38, NF-κB
Chae *et al.* [88]	*Hylomecon hylomeconoides*	Ethanol extracts	↓NO, IL-6 , ERK1/2, p38
Debnath *et al.* [89]	*Chrysanthemum indicum*	Flower	Antioxidant activity

IL: Interleukin; NF-κB: Nuclear factor κB; IFN: Interferon; NO: Nitric oxide; TNF-α: Tumor necrosis factor α; iNOS: Inducible nitric oxide synthase; Cox: Cyclooxygenase, ERK: Extracellular signalregulated kinase, JNK: c-Jun NH2-terminal kinase; PGE2: Prostaglandin E2.
